# Identification and characterization of *Xenopus tropicalis* common progenitors of Sertoli and peritubular myoid cell lineages

**DOI:** 10.1242/bio.019265

**Published:** 2016-07-27

**Authors:** Tereza Tlapakova, Thi Minh Xuan Nguyen, Marketa Vegrichtova, Monika Sidova, Karolina Strnadova, Monika Blahova, Vladimir Krylov

**Affiliations:** 1 Charles University in Prague, Faculty of Science, Vinicna 7, Prague 2 128 44, Czech Republic; 2 Laboratory of Gene Expression, Institute of Biotechnology, Academy of Sciences of the Czech Republic, Videnska 1083, Prague 4 142 20, Czech Republic

**Keywords:** Testicular somatic cells, *Xenopus tropicalis*, Migration potential, Common progenitor

## Abstract

The origin of somatic cell lineages during testicular development is controversial in mammals. Employing basal amphibian tetrapod *Xenopus tropicalis* we established a cell culture derived from testes of juvenile male. Expression analysis showed transcription of some pluripotency genes and Sertoli cell, peritubular myoid cell and mesenchymal cell markers. Transcription of germline-specific genes was downregulated. Immunocytochemistry revealed that a majority of cells express vimentin and co-express Sox9 and smooth muscle α-actin (Sma), indicating the existence of a common progenitor of Sertoli and peritubular myoid cell lineages. Microinjection of transgenic, red fluorescent protein (RFP)-positive somatic testicular cells into the peritoneal cavity of *X. tropicalis* tadpoles resulted in cell deposits in heart, pronephros and intestine, and later in a strong proliferation and formation of cell-to-cell net growing through the tadpole body. Immunohistochemistry analysis of transplanted tadpoles showed a strong expression of vimentin in RFP-positive cells. No co-localization of Sox9 and Sma signals was observed during the first three weeks indicating their dedifferentiation to migratory-active mesenchymal cells recently described in human testicular biopsies.

## Abstract (precis)

**Summary:** We identified cells co-expressing differentiation markers of Sertoli and peritubular myoid cell lineages in *X. tropicalis* through the establishment and characterization of cell culture derived from juvenile testis.

## INTRODUCTION

The architecture of seminiferous tubules is tightly associated with the presence of peritubular myoid cells (PTMC) and Sertoli cells (SC) both forming basement membranes underlying the seminiferous epithelium (Skinner et al., 1985). Sertoli cells (SC) are also indispensable for germ cell maturation and differentiation (Berndtson and Thompson, 1990; Johnson et al., 1984). They stretch to the lumen and have an intimate contact with developing gametes ranging from spermatogonia located on the base to spermatids in the centre. Sertoli cells also provide a signalling niche via expression of several growth factors and cytokines (De Rooij, 2009). In addition, with PTMCs they participate in the formation of seminiferous cords and appropriate vascularization through the expression of Sry (Koopman et al., 1990) and downstream signalling cascades (Bott et al., 2006). Moreover, they function as an immunological barrier since testes are an immunologically privileged organ (Dufour et al., 2005). Leydig cells start to differentiate in the end of the proliferative phase of Sertoli cells (Baker et al., 1999; Nef et al., 2000; O'Shaughnessy et al., 2008) and form the stable cell line indispensable for the production of male sex hormones.

The origin of individual testicular somatic cell lineages in mouse is still controversial. Precursors of Sertoli cells were detected in the population of coelomic epithelial cells migrated into the gonad 11.5 days post-coitum (Karl and Capel, 1998). Later publications disproved these findings and showed that pre-Sertoli cells are already present in the developing gonad together with arrived germ cells and form Sertoli germ cell mass (SGCM) (reviewed in Cool et al., 2012). Based on the expression of the low affinity neurotrophin receptor p75, peritubular myoid cells were found as mesenchymal precursors migrated from an adjacent mesonephric tissue (Campagnolo et al., 2001); however, this result was also disproved and only endothelial cells, but not PTMCs, were identified as a migrating population from mesonephros to the gonadal base (Combes et al., 2009). Authors performed *ex vivo* assay in which they co-cultured a wild-type male genital ridge alongside mesonephroi constitutively expressing GFP (Nishino et al., 2000). They found that endothelial cells with VE-cadherin expression, and not p75 positive PTMCs, are the only migrating cells entering the gonad. Furthermore, endothelial cells were identified as being indispensable for establishing a proper seminiferous tubule architecture (Combes et al., 2009).

Regarding humans, Chikhovskaya et al. (2012) used frozen testicular biopsies for variable enzymatic digestions and subsequent cultivation *in vitro.* Over 30-50 days embryonic stem cell (ESC)-like colonies emerged. Gene expression analysis revealed a low level of pluripotency markers such as *POU5F1*, *NANOG* and *SOX2* which was in disagreement with similar studies performed on mouse where such colonies were found to be derived from dedifferentiated spermatogonial stem cells (SSCs) and showed the ability to form teratoma (Guan et al., 2006; Kanatsu-Shinohara et al., 2004, 2008; Ko et al., 2009). Human testicular cells expressed mesenchymal stem cell (MSC) markers and were able to differentiate to three mesodermal lineages (adipocytes, chondrocytes and osteocytes) indicating their multipotent but not pluripotent character (Chikhovskaya et al., 2014).

So far the majority of experiments employing testicular cells have been conducted in mammalian models; however, studies of their migration and differentiation potential *in vivo* via transplantation into early embryos are hampered by the inner embryonic development in the womb. In addition, Sertoli cells are able to survive after xenogeneic transplantation into the evolutionarily distant host. This feature is interesting for basic research in the field of evolutionary immunology due to the potential utilization of xenogeneic Sertoli cells for co-transplantation with grafts without the need of immunosuppressive treatment. In this regard, well-established non-mammalian vertebrate model organisms are desirable and the diploid amphibian *Xenopus tropicalis* suits these requirements well. *X. tropicalis* is highly valuable in the fields of early vertebrate development, cell biology, and genome evolution, and large oocytes, outer fecundation and embryonic development make it feasible for microinjection or transplantation experiments. The *X. tropicalis* genome is fully sequenced and arranged into linkage groups (Hellsten et al., 2010; Wells et al., 2011), compared to evolutionarily-close fish model organisms (zebrafish, carp, trout etc.) the genome is diploid (Tymowska, 1973) and thus more suitable for gene function studies (Geach and Zimmerman, 2011).

Here we present a successful establishment and *in vitro* and *in vivo* (allogeneic transplantation into the tadpole peritoneal cavity) characterization of a stable cell culture derived from mechanically disrupted testes of a juvenile *X. tropicalis* male three months after metamorphosis. The cell culture is composed of a proliferative testicular cell feeder layer [*X. tropicalis* testicular somatic cells (XtTSC)] and testicular cell colonies [*X. tropicalis* testicular somatic cell colonies (XtTSCc)]. Reverse transcription (RT) and quantitative polymerase chain reaction (qPCR) analysis revealed a strong expression of mesenchymal, Sertoli and peritubular myoid cell markers; however germ cell markers were not detected, which confirms their somatic origin. Double immunocytochemical staining against Sox9 (SC marker) and Sma (marker of PTMC) clearly showed the presence of both antigens in ∼80% of cells. This result indicates that at least in *Xenopus* there exist a common progenitor of Sertoli cell and PTMC lineages emerging from mesenchymal cells present in developing testes.

## RESULTS

### Morphological and gene expression characterization of *X. tropicalis* testicular cell culture

**Fig. 1.**
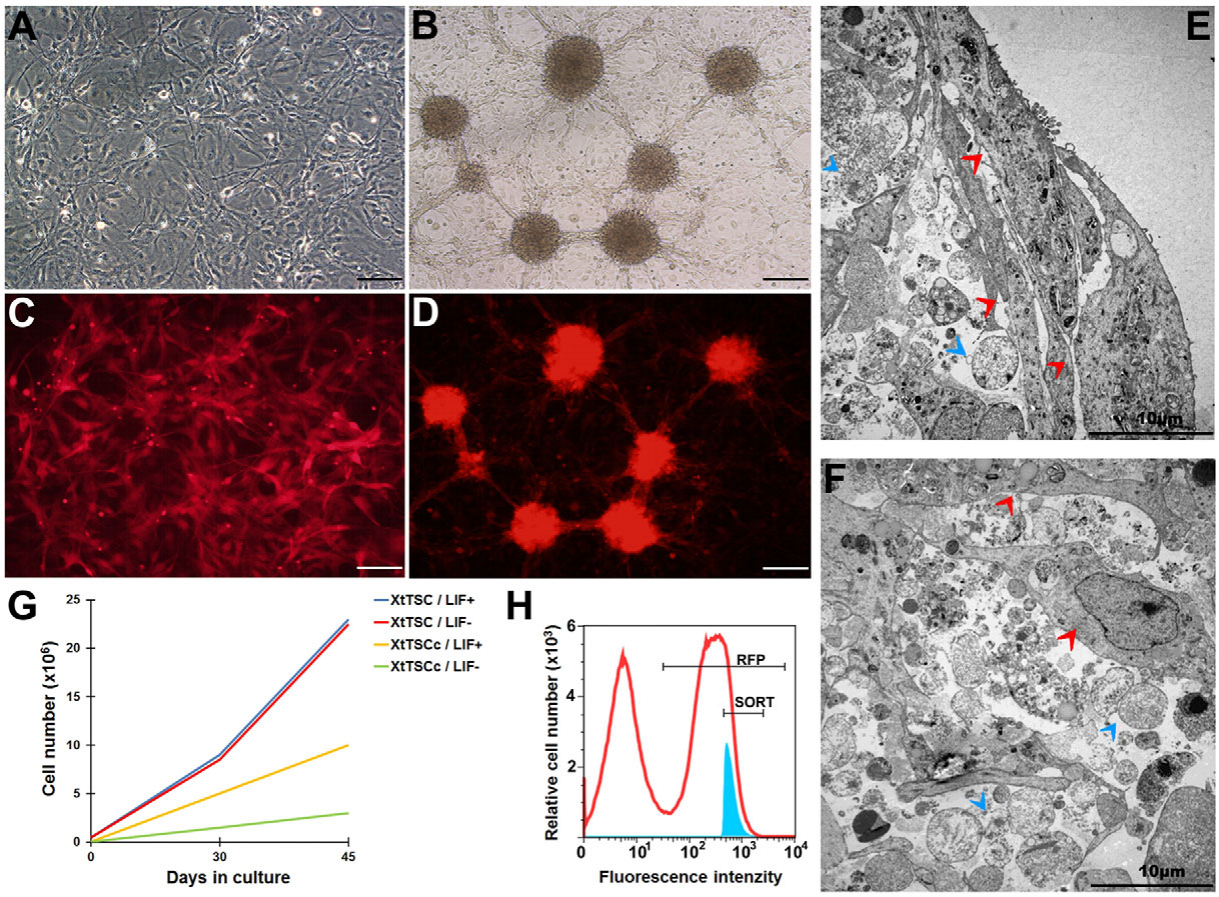
***In vitro* characterization of *X. tropicalis* cell culture.** (A,B) Testicular somatic cell culture in morphology of adherent feeder layer (XtTSC) (A) and after long-term cultivation which enables the forming of colonies (XtTSCc) (B). (C) *X. tropicalis* transgenic XtTSC expressing Katushka RFP under CAG promotor (XtTSC-RFP). (D) Transgenic Katushka RFP expressing XtTSC in colonies (XtTSCc-RFP). (E,F) Structure of *in vitro* testicular cell colony visualized by TEM. In the colony the cells are placed in an extensive amount of extracellular matrix with two or three tight layers of XtTSCs surrounding the colony at the edge (E). Both XtTSC and XtTSCc are present in the centre of the colony (F). The XtTSCc are clearly several times smaller than the XtTSC. Red arrowheads, XtTSC; blue arrowheads, XtTSCc. (G) *X. tropicalis* cell culture proliferation during long-term cultivation in medium with or without recombinant mouse LIF. (H) Representative graph of FACS sorting after nucleofection. Only ∼15% of living transgenic cells with the highest intensity of fluorescent signal were sorted (blue area). Scale bars A,C:100 μm; scale bars B,D: 200 μm; scale bars E,F: 10 μm.

After establishing a *X. tropicalis* testicular cell culture, the adherent cells formed a feeder layer (XtTSC) with the morphological characteristics of Pre-Sertoli cells (Fig. 1A). Long-term cultivation enables the forming of colonies (XtTSCc) resembling embryonic stem cells (ESC) (Fig. 1B). The ultrastructure and cell arrangement within the colony were visualized via transmission electron microscopy (TEM). Sertoli cell-like cells surrounded the colony in two or three tight layers (Fig. 1E), and few of them were found inside. TEM showed that XtTSCs and XtTSCcs were arranged individually in an extensive amount of extracellular matrix (Fig. 1F).

**Fig. 2.**
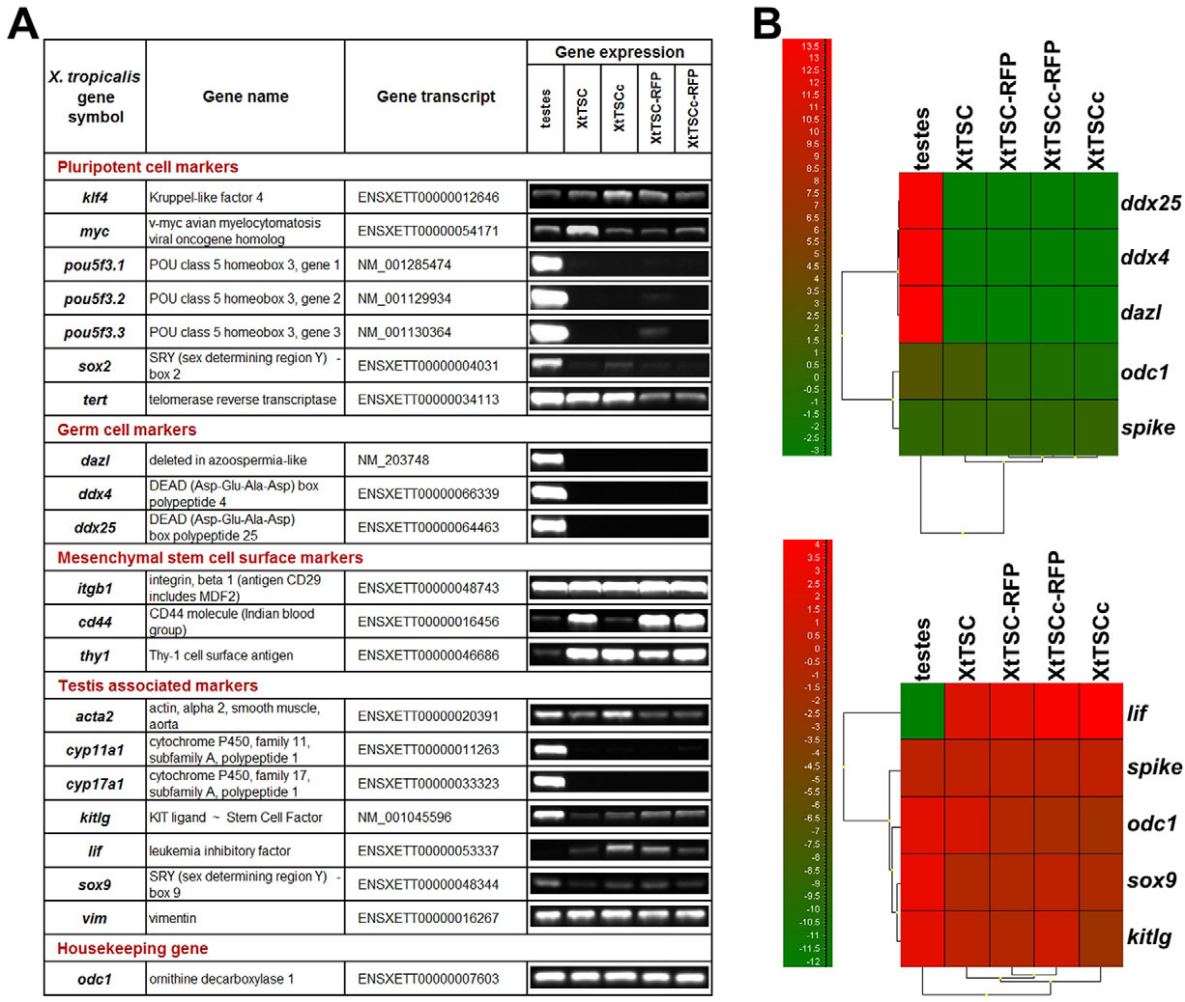
**Expression analysis of *X. tropicalis* testicular culture.** (A) RT-PCR analysis of *X. tropicalis* testis, XtTSC, XtTSCc, XtTSC-RFP and XtTSCc-RFP. (B) Hierarchical clustering presented as a qPCR heatmaps of germ cell markers and selected testis associated markers. A scale of colours indicates level of expression (the highest expression is shown by bright red, whereas the lowest expression is shown by bright green). Similarity between cell types/genes is indicated by the height at which the dendrograms are joined. The RNA spike represents a highly stable transcript across the cell types and odc1 represents housekeeping gene.

Reverse transcription polymerase chain reaction (RT-PCR) analysis revealed a similar gene expression profile for both cell types. The feeder layer and colonies were positive for pluripotency markers *klf4* (kruppel-like factor 4), *c-myc* and telomerase reverse transcriptase (*tert*). However, the key pluripotency genes *POU5F1* (in *X. tropicalis pou5f3.1*, *pou5f3.2* and *pou5f3.3*) (Morrison and Brickman, 2006; Frankenberg et al., 2014) or *sox2* (sex determining region Y box 2) were downregulated, suggesting that our cells are not in a pluripotent state as has been defined in the mouse model. Unfortunately, expression of the *nanog* gene, another key player in pluripotency acquisition (Silva et al., 2009), could not be determined since no homologue has been described in *Xenopus* yet. Germ cell markers such as *dazl*, *ddx4* and *ddx25* were not detected, this result confirmed the somatic origin of testicular cells. More detailed characterization was based on expression markers encompassing Sertoli cells (*sox9*, *kitlg*, *vim* and *lif*), peritubular myoid cells (*acta2* and *lif*), Leydig cells (*cyp11a1* and *cyp17a1*) and markers of mesenchymal cells (*itgb1*- *cd29*, *cd44* and *thy1*-*cd90*). Except for Leydig cell markers, both cell types were positive for all above mentioned genes (Fig. 2A).

**Fig. 3.**
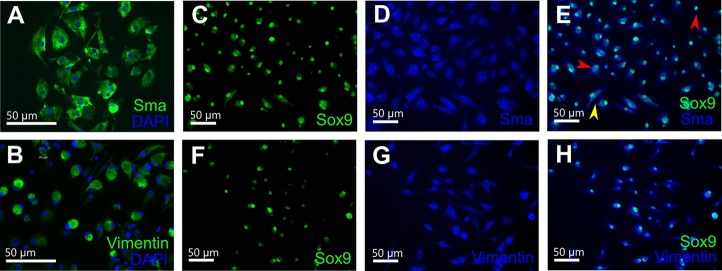
**Immunocytochemistry of *X. tropicalis* testicular cell culture expressing Katushka RFP.** Positive staining for Sma (marker of peritubular myoid cells, green) (A) and vimentin (marker of mesenchymal cells and peritubular myoid cells, green) (B). Nuclei were counterstained with DAPI (blue). (C-E) Double staining with Sox9 (marker of Sertoli cells, green) (C) and Sma (blue) (D) antibodies. (E) Merge of C and D. Yellow arrowhead, cell expressing both antigens; red arrowheads, cells expressing only Sox9 or only Sma. (F-H) Double staining with Sox9 (green) (F) and vimentin (blue) (G) antibodies. (H) Merge of F and G. Scale bars: 50 μm.

**Fig. 4.**
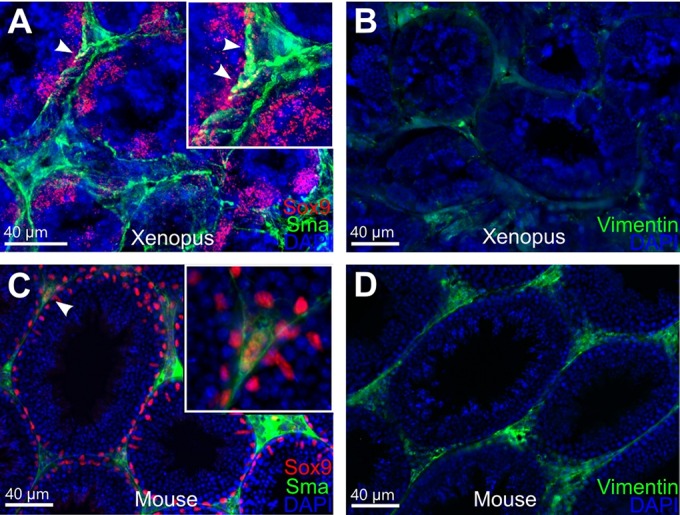
**Immunohistochemistry of agarose embedded testicular sections from *X. tropicalis* and mouse adult males.** (A,C) Double staining with Sox9 (red) and Sma (green) antibodies. White arrowheads indicate potential common precursor cells for Sertoli and PTM cell lineages expressing both antigens in *X. tropicalis* (A) and mouse (C) samples. Insets show a detailed view of structures marked with white arrowheads in underlying figures. (B,D) Staining with vimentin (green) antibody on *X. tropicalis* (B) and mouse (D) samples. Nuclei were counterstained with DAPI (blue). Scale bars: 40 μm.

RT-PCR data was confirmed by qPCR analysis. The standard deviation of the RNA spike quantification across all samples was 0.2 cycles, which shows evidence of minimal technical variation and high reproducibility. The hierarchical clustering was performed according to two groups of analyzed markers, germ cell markers and testis-associated markers. Each heatmap included an RNA spike as a highly stable transcript across the cell types and housekeeping gene *odc1*. The result of the clustering indicated that the gene expression profile of testicular tissue is different from XtTSC and XtTSCc groups. Transcripts *dazl*, *ddx25* and *ddx4* are exclusively expressed in the testes, whereas expression of *lif* is substantially reduced in comparison with XtTSCs and XtTSCcs (Fig. 2B). Immunocytochemistry on feeder cells and colonies employing Sox9, Sma (smooth muscle α-actin) and vimentin antibodies revealed their colocalization on more than 80% of cells (Fig. 3). Taken together, we concluded that *X. tropicalis* testicular cell culture represents a population of Sertoli cell and PTMC common progenitors. To test if these cells are also present in adult individuals we prepared agarose embedded sections of *X. tropicalis* and mouse testes. After double staining with Sox9 and Sma antibodies we found individual cells expressing both antigens in the interstitial space close to the seminiferous tubules in *X. tropicalis* and even in mouse testis (Fig. 4).

Sertoli cells produce many soluble factors necessary for germ cell survival and proliferation. One of them, the leukemia inhibitory factor (Lif), turned out to be crucial for *in vitro* enhanced XtTSC survival and colony forming activity. Although both the XtTSCs and XtTSCcs express their own *lif* (measured on the RNA level) (Fig. 2A), the addition of recombinant mouse LIF into the cultivation medium entailed a rapid formation and expansion of XtTSCc colonies. However, a total proliferation rate was unaffected by LIF since experimental groups (+LIF and −LIF) revealed the same growth curves as depicted on Fig. 1G.

### *In vivo* migration potential of testicular somatic cells

**Fig. 5.**
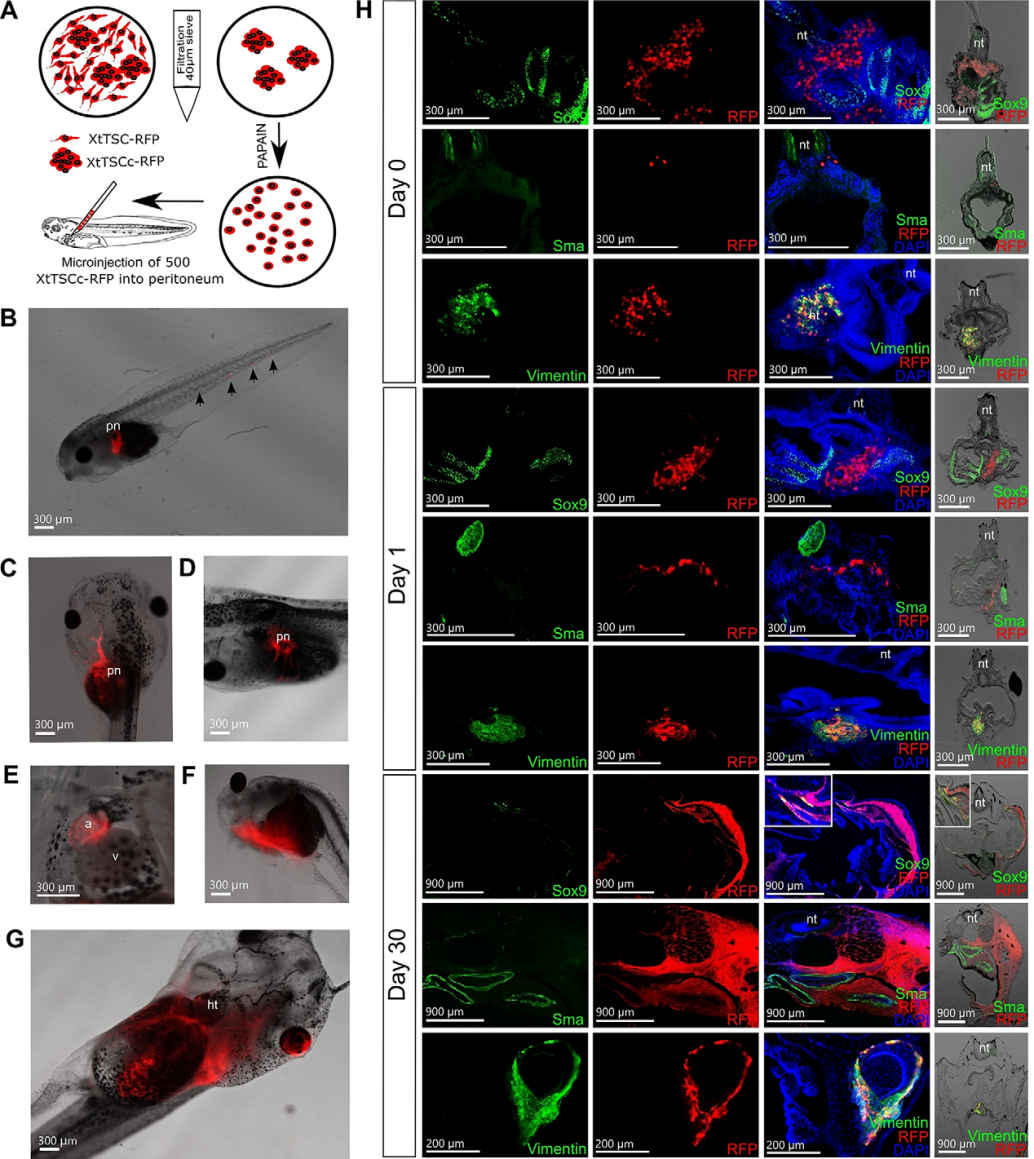
**Migration potential of *X. tropicalis* testicular somatic cells after allogeneic transplantation into peritoneal cavity of tadpoles in stage 41.** (A) Scheme of XtTSCc-RFP preparation prior to transplantation employing isolation of cell colonies using 40 μm sieve and subsequent single cell dissociation with papain. 500 cells were microinjected dorsally into peritoneal cavity. (B-G) Observation of RFP-positive cells in transplanted tadpoles under stereo microscopy. (B) Cell deposit in pronephros and tail 1 day after microinjection. (C,D) Cell deposits in pronephros 13 days after microinjection. (E) Migration of RFP-positive cells into heart atrium 15 days after transplantation. (F,G) Cell-to-cell net growing through the tadpole body observable 30 days after microinjection. (H) Immunohistochemistry of agarose-embedded sections of transplanted tadpoles using antibodies against Sox9, Sma and vimentin (green) and Katushka RFP (red) 0, 1 and 30 days after transplantation. RFP-positive cells were stained with vimentin antibody but not Sox9 or Sma even 2 h after transplantation. 30 days after microinjection, few cells start to express Sox9 indicating potential redifferentiation into Sertoli cells or chondrocytes where Sox9 is considered as a specific marker. The first three figures in each line were taken under fluorescence microscopy. The figures on the right side were taken under fluorescence stereomicroscopy. Nuclei were counterstained with DAPI (blue). Scale bars B-G, H (0 and 1 day after transplantation), 300 μm; scale bars in H (30 days after transplantation, staining with Sox9 and Sma antibodies), 900 μm; scale bars in H (30 days after transplantation, staining with vimentin antibody), 200 μm.

A peritoneal cavity of tadpoles at stage 41 was used for the transplantation of transgenic XtTSCc-RFP and XtTSC-RFP expressing Katushka RFP under ubiquitous CAG promotor (Fig. 1C,D). Cell microinjection of 500 cells per peritoneum was performed through the dorsal side as depicted in (Fig. 5A). One week after transplantation we observed cell deposits mostly in heart and pronephros (Fig. 5B-E). During three following weeks transplanted cells strongly proliferated and formed a dense cell-to-cell connecting net growing through the tadpole's body (Fig. 5F,G). Immunohistochemical analysis of agarose embedded sections of tadpoles 0, 1 and 30 days after transplantation revealed a strong vimentin and RFP colocalization (Fig. 5H). However, expression of Sox9 and Sma, found in testicular cells prior to microinjection was not detected even 2 h after transplantation when tadpoles from group ‘0 day’ were fixed. Interestingly, 30 days after microinjection we observed Sox9 expression in a few RFP-positive cells indicating their potential differentiation into Sertoli cells or chondrocytes where this protein is also considered a cell-specific marker. Since both cell types (XtTSCc-RFP and XtTSC-RFP) showed the same migration and expression pattern, here we published only data concerning transplantation of testicular somatic cell colonies (XtTSCc).

## DISCUSSION

In this study we characterized a newly established *X. tropicalis* testicular cell culture encompassing adherent feeder Sertoli-like cells (XtTSC) and cell colonies resembling ESC (XtTSCc). A long-term stem cell culture derived from testis was firstly described by Kanatsu-Shinohara et al. (2003) in mouse. Testes from newborn males were enzymatically dispersed and transferred to gelatine-coated plates. Here, in the presence of the glial cell line-derived neurotrophic factor (GDNF), epidermal growth factor (EGF), fibroblast growth factor 2 (FGF2) and leukemia inhibitory factor (LIF) spermatogonia could be propagated as cell clumps. One year later the same team announced a conversion of germ cells (GS) to multipotent germ stem cells (mGSs) using standard ESC cultivation medium containing 15% fetal bovine serum (FBS) and LIF (Kanatsu-Shinohara et al., 2004). In humans, the establishing of similar cell lines was found as a controversial. Four papers described the existence of ES-like cell colonies derived from testicular tissue (Conrad et al., 2008; Golestaneh et al., 2009; Kossack et al., 2009; Mizrak et al., 2010); however, in three of them (Golestaneh et al., 2009; Kossack et al., 2009; Mizrak et al., 2010) authors failed to induce teratoma after subcutaneous transplantation of testicular stem cells into immunodeficient mice. Chikhovskaya et al. (2012) revised previously published data of Mizrak et al. (2010) and performed an expanded study on human testis embryonic stem cell-like cells (htES-like cells) derived from frozen testicular samples using different enzyme digestion and cultivation conditions. Gene expression analysis revealed an expression of *KLF4* and *MYC* but not *SOX2*, *NANOG* or key pluripotency marker *POU5FI* indicating their multipotent rather than pluripotent character. RT-PCR confirmed the presence of *CD73*, *CD90* and *CD150* and absence of *CD31*, *CD34* and *CD45* surface markers characteristic for the expression profile of mesenchymal stem cells. Repeated efforts to induce teratoma with htES-like cells in immunodeficient mouse failed. Chikhovskaya et al. (2014) differentiated htES-like cells *in vitro* into three mesodermal cell lineages typical for mesenchymal stem cells (adipocytes, chondrocytes and osteocytes). In this study, up to five days after the mechanical disruption of *X. tropicalis* testis in the culture medium, adherent cells migrating from the organ were observed. Two months later small colonies of ES-like cells (XtTSCc) started to appear on the feeder layer (XtTSC). RT-PCR analysis showed the same expression profile as in the case of htES-like cells (Chikhovskaya et al., 2012). We detected only some pluripotency markers (*klf4* and *myc*) and markers of mesenchymal stem cells *cd29* (*itgb1*), *cd44* and *cd90* (*thy1*). Germ cell markers (*dazl*, *ddx4* and *ddx25*) were downregulated which further confirms the somatic rather than germ line origin of our testicular cells. Unlike Chikhovskaya et al. (2012) we performed gene expression analysis regarding testicular somatic lineages: Sertoli cells, peritubular myoid cells and Leydig cells. Except for the Leydig cell markers (*cyp11a1 and cyp17a1*) we found positive reactions for both remaining cell types (*sox9*, *kitlg*, *lif*, *acta2* and *vim*). Immunocytochemistry revealed a colocalization of nuclear Sox9 and cytoplasmic Sma antigens. Together with previously published expression data in humans, we concluded that in *X. tropicalis* there exists a common progenitor of Sertoli and PTM cell lineages with morphological and expression characteristics of mesenchymal stem cells. Stem cell precursors for Leydig cells were already identified and characterized in interstitial space of rat testis close to seminiferous tubules (Inoue et al., 2016; Shan and Hardy, 1992; Stanley et al., 2011). We observed the same localization of cells double stained with Sox9 and Sma antibodies in *X. tropicalis* agarose embedded testicular sections and even in mouse samples.

The growth curve of our amphibian testicular cells showed a strong correlation between the addition of mouse leukemia inhibitory factor (mLIF) to the cultivation medium and cell colony forming activity. RT-PCR analysis revealed a relatively high *lif* transcription in both cell types (XtTSC and XtTSCc). In testis, a LIF production was determined in PTM cells located between the seminiferous tubules and the interstitium (Piquet-Pellorce et al., 2000) and also in remaining somatic cell types (Sertoli and Leydig cells) and spermatogonia (Jenab and Morris, 1998). LIF has an effect on spermatogonia proliferation and on the increased survival rate of Sertoli cells (De Miguel et al., 1996). We observed that for a successful establishment of amphibian testicular cell culture and its colony-forming activity, at least the initial addition of mLIF to the cultivation medium is indispensable. Further supplementation is important for the colony forming activity, but not for the testicular cell proliferation and survival. It is possible that initial addition of mouse LIF triggers the production of *Xenopus* homolog by testicular cells which is sufficient for their maintenance in the cell culture, but not for the formation of cell colonies.

The conservation of mammalian and non-mammalian Lif amino acid sequences is rather low (20-40%); however, all orthologous proteins share a conserved three-dimensional structure (Mathieu et al., 2012). As for lower vertebrates, *lif* cDNA was cloned in zebrafish, carp and goldfish (Fujiki et al., 2003; Abe et al., 2007; Hanington and Belosevic, 2007). Morpholino-based knockdown of *lif* in zebrafish revealed no obvious effect on early embryonic development. However, when its receptor (LIFR) had been targeted, effects on proper neural development were observed (Hanington et al., 2008). In chicken, LIF has been shown to function as an anti-differentiation factor for blastoderm cells (Horiuchi et al., 2006). In amphibians, its effect on early embryonic development is still unknown.

Unlike testicular cell culture, RT-PCR and qPCR analysis showed a low expression of *lif* in adult testes. As mentioned above, in mouse, LIF is mostly produced by peritubular cells located on a periphery of seminiferous tubules (Piquet-Pellorce et al., 2000). It is possible, that in *Xenopus* testis Lif is expressed by scarce cells positively stained for Sox9 and Sma antigens. When transferred out of the testicular niche, these cells can proliferate *in vitro* and produce a higher amount of Lif.

El Jamil et al. (2008) studied the distribution of *sox9* mRNA and protein in *X. tropicalis* testicular and ovarian cryosections. In males, authors observed a Sox9 expression in nuclei of supporting (pre-Sertoli) cells located on the base of seminiferous tubules in the vicinity of germ cells. Unlike higher vertebrates, Sox9 is also expressed in oocyte cytoplasm indicating its role in the testicular differentiation but not in the sex determination.

To study of migration potential of *X. tropicalis* testicular somatic cells we performed a series of transplantation experiments with transgenic cell culture expressing Katushka RFP (XtTSCc-RFP). Cells were microinjected into the peritoneal cavity of tadpoles at stage 41. During the first week after microinjection we observed migration of RFP-positive cells into heart, pronephros (tadpole kidney) and intestine. The same organs are also major migratory targets for mouse mesenchymal stem cells intravenously injected into the bloodstream (reviewed in Cornelissen et al., 2015). Strong expression of vimentin in transplanted cells observed even one month after microinjection is typical for migratory mesenchymal cells (reviewed in Kim et al., 2014). In addition, differentiation markers of Sertoli and PTM cells (Sox9 and Sma) were downregulated indicating a dedifferentiation process towards mesenchymal stem cells able to successfully proliferate and migrate through the tadpole body.

## MATERIALS AND METHODS

### Ethical statement

This study was carried out in strict accordance with the Act No. 246/1992 Coll., on the protection of animals against cruelty. An official permission was issued to the Faculty of Science, Charles University in Prague by the Ministry of Education, Youth and Sports of the Czech Republic (No. MSMT-37376/2014-4, date of expiry 3. 3. 2019).

### *X. tropicalis* testicular somatic cell culture

The *X. tropicalis* testicular somatic cell culture was established from testes of juvenile male (Ivory Coast strain) 6 months after metamorphosis. For wash steps, diluted PBS (2:1 PBS/deionized H_2_O) was used due to different cell osmolarity of amphibian contrary to mammalian cells. Testes were extensively washed with diluted PBS and disrupted with needles in cultivation medium consisting of 33.3% L-15 and 33.3% RPMI 1640 HEPES modification medium (both Sigma-Aldrich) supplemented with 10% FBS (Life Technologies), 1.33 mg/ml sodium bicarbonate, 2 mM L-glutamine and 50 µg/ml gentamicin (all Sigma-Aldrich). Testicular explants were cultivated at 29.5°C with 5.5% CO_2_ for 5 days without any interference. Preparation of primary culture was successfully repeated three times with different *X. tropicalis* individuals originated from various breedings. All three lines exhibited the same morphology and behaviour during long-term cultivation and subsequent experiments.

For XtTSCc cultivation, medium has been improved with 1 mM sodium pyruvate, 0.1 mM 2-mercaptoethanol (both Sigma-Aldrich) and 1000 U/ml recombinant mouse LIF (ESGRO; Millipore) according to Chowdhury et al. (2010). The XtTSCc medium was changed every three days and cells were passaged every two weeks. To obtain a single cell suspension of XtTSCc, dissociation by a papain solution (61.25 mg/l papain, 0.5 mM EDTA and 1 mM L-cysteine in PBS without Ca2+, Mg2+; Biochrom AG) was efficient. Disintegration of colonies using Accutase™ (Thermo Electron Corporation), Biotase (Biochrome AG) or trypsin-EDTA (0.5% trypsin-0.2% EDTA) was always incomplete. To measure growth, viable cells were seeded at a density of 1×10^5^ cells per flask (75 cm^2^) and cultured in XtTSCc medium with and without recombinant mouse LIF for 45 days. During this time period cells were counted three times (15 days interval). Mean values were used to plot a growth curve for both cell types together and for XtTSCc separately.

### Visualization of XtTSCc via transmission electron microscopy (TEM)

XtTSCc was separated from XtTSC feeder layer by trypsin-EDTA treatment and filtered through a 20 µm filter (CellTrics^®^, Partec). For TEM, colonies were fixed, dehydrated in a graded ethanol series and acetone, and embedded in Araldite 502/PolyBed 812 resin (Polyscience, Inc.) as previously described (Hyliš et al., 2007). Ultrathin sections (70 µm) were stained with uranyl acetate and lead citrate and examined with the JEOL 1011 transmission electron microscope with a Veleta CCD camera and Olympus Soft Imaging Solution GmbH software.

### Preparation of transgenic Katushka RFP testicular cell culture

Testicular cells were electroporated with 6 μg of ISpBSIISK-CAG-Katushka RFP vector (Shcherbo et al., 2007) using Nucleofector™ 2b Device (Lonza), program T-020 and nucleofection solution (5 mM KCl, 15 mM MgCl_2_, 50 mM Na_2_HPO_4_, 100 mM NaCl). One month after nucleofection, transfected cells were separated on the basis of Katushka RFP signal by a fluorescence-activated cell sorting (FACS) using the inFlux v7 Sorter (BD Bioscience) (Fig. 1H).

### RT-PCR and qRT-PCR

Total RNA was isolated from the adult *X. tropicalis* testes, XtTSC, XtTSCc, XtTSC-RFP and XtTSCc-RFP using RNeasy Mini Kit (Qiagen) according to the manufacturer's instructions. Reverse transcription was performed with the same amount of total RNA (200 ng) by the RevertAid H Minus First Strand cDNA Synthesis Kit (Thermo Scientific). The relative expression of target genes was determined by using *odc1* as a reference gene. Quantitative RT-PCR (qRT-PCR) was performed with a real-time CFX384 cycler system (BioRad) using iQTM SYBR^®^ Green Supermix (BioRad). A RNA spike (TATAA Biocenter) was used to validate the reverse transcription and the quantitative PCR reactions. The detailed protocol of cDNA synthesis and qPCR reaction was already described (Flachsova et al., 2013; Sidova et al., 2015). For primer sequences and further details, see the Table S1.

### Transplantation of testicular somatic cells into tadpole's peritoneal cavity

*X. tropicalis* embryos were produced by the standard *in vitro* fertilization procedure (Geach and Zimmerman, 2011). Embryos were cultivated in 0.05×MMR with gentamicin (50 µg/ml) for two days (stage 41). The developmental stage was determined according to Nieuwkoop and Faber (1994). Katushka RFP-positive testicular cells were detached from the bottom of the cultivation flask by trypsin. XtTSCc were separated using 40 μm sieve and colonies were then disintegrated to single cell suspension by papain. 40 nl containing 500 Katushka RFP-positive cells was microinjected into each peritoneal cavity of tadpoles at stage 41 using a thin glass capillary (Drummond, type 1-000-0500) and the Narishige IM-300 pneumatic microinjector (Fig. 5A). To prevent tadpole movements, the microinjection experiments were performed in an agarose-coated Petri dish filled with 0.05× MMR containing few drops of 0.02% MS222 (Sigma-Aldrich). After transplantation, tadpoles were cultivated for up to one month and the distribution of RFP positive cells was observed under a fluorescence stereomicroscope (Olympus).

### Immunohistochemistry of vibratome sections from transplanted tadpoles

Transplanted tadpoles at stage 41 (day 0), 45 (day 1) and 55 (day 30) were fixed overnight in MEMFA (0.1 M MOPS, 2 mM EGTA, 1 mM MgSO4, and 3.7% formaldehyde) at 4°C. Agarose embedding was performed by gradual rehydration using 90, 75, 50 and 25% methanol diluted by PBST (PBS plus 0.1% Tween 20), following by 3 times washing with PBS. Tadpoles were then immersed into 3% agarose in PBS overnight at 48.5°C and cooled down. Agarose blocks with fixed tadpoles were then cut into 30-40 µm sections on vibratome (Leica 1200) in PBS. The sections were permeabilized with 0.1% Triton X-100 in PBS for 1 h and blocked with TNB [0.1 M Tris-HCl, 0.15 M NaCl, 0.5% Blocking Reagent (Boehringer Mannheim GmbH)] for the same time. Incubation with primary antibody in TNB was done for 3 days at 4°C. The dilution of primary antibodies against vimentin, Sox9 and Sma was 1:40, 1:300 and 1:400 respectively or 1:5000 for anti-tRFP (rabbit, Evrogen). Appropriate secondary antibody (Sigma) was applied for 2 h at room temperature following washing five times with PBSTr. Individual sections were mounted on slides with Mowiol/DAPI mounting medium and observed under fluorescence microscopy. For details of antibodies used in immunofluorescence, see the Table S2.
